# Causal associations of ischemic stroke, metabolic factors, and related medications with epilepsy: a Mendelian randomization study

**DOI:** 10.3389/fneur.2024.1464984

**Published:** 2024-11-13

**Authors:** Wencai Wang, Menghao Liu, Fengling Liu, Zun Wang, Wei Ye, Xianfeng Li

**Affiliations:** The Second Affiliated Hospital of Harbin Medical University, Harbin, China

**Keywords:** epilepsy, Mendelian randomization, ischemic stroke, metabolic factors, medications

## Abstract

**Background:**

Earlier researches have demonstrated that ischemic stroke, metabolic factors, and associated medications may influence the risk of epilepsy. Nevertheless, the causality between these elements and epilepsy remains inconclusive. This study aims to examine whether ischemic stroke, metabolic factors, and related medications affect the overall risk of epilepsy.

**Methods:**

We used single nucleotide polymorphisms associated with ischemic stroke, hypothyroidism, hypertension, blood glucose levels, high cholesterol, serum 25-Hydroxyvitamin D levels, testosterone, HMG CoA reductase inhibitors, and beta-blocking agents as instrumental variables in a Mendelian randomization technique to investigate causality with epilepsy. Multiple sensitivity methods were performed to evaluate pleiotropy and heterogeneity.

**Results:**

The IVW analysis revealed positive associations between ischemic stroke (OR = 1.29; *p* = 0.020), hypothyroidism (OR = 1.05; *p* = 0.048), high blood pressure (OR = 1.10; *p* = 0.028), high cholesterol (OR = 1.10; *p* = 0.024), HMG CoA reductase inhibitors (OR = 1.19; *p* = 0.003), beta-blocking agents (OR = 1.20; *p* = 0.006), and the risk of epilepsy. Conversely, blood glucose levels (OR = 0.79; *p* = 0.009), serum 25-Hydroxyvitamin D levels (OR = 0.75; *p* = 0.020), and testosterone (OR = 0.62; *p* = 0.019) exhibited negative associations with the risk of epilepsy. Sensitivity analyses confirmed the robustness of these findings (*p* > 0.05).

**Conclusion:**

Our research suggests that ischemic stroke, hypothyroidism, high blood pressure, high cholesterol, HMG CoA reductase inhibitors, and beta-blockers may increase the risk of epilepsy, whereas serum 25-Hydroxyvitamin D levels and blood glucose levels may reduce the risk.

## Introduction

Epilepsy is a common neurological condition caused by highly synchronized neuronal discharges (1). Its etiology is complex, encompassing structural, genetic, infectious, metabolic, immunological, and other unknown factors (2, 3). The disorder significantly impacts global physical health, particularly among infants and the elderly, leading to 13 million injuries and disabilities annually (4). Therefore, identifying the underlying causes is crucial for the effective management of epilepsy.

Numerous studies have shown that ischemic stroke (5), and metabolic factors such as hypothyroidism (6), hypertension (7, 8), blood glucose levels (9), high cholesterol (10), serum 25-Hydroxyvitamin D levels(25(OH)D) (11), and testosterone (12) are associated with the development of epilepsy. Additionally, certain medications, including HMG CoA reductase inhibitors(HMGCR) (13) and *β*-blockers (14) have been discovered to raise the risk of epilepsy. However, the causal relationship between these hazard elements and epilepsy remains unclear, as most previous studies were observational and yielded inconsistent findings.

Mendelian randomization (MR) is an epidemiological technique that uses genome-wide association study (GWAS) data to investigate the causality between different phenotypes and diseases (15). Consequently, in our research, we employed MR to assess the underlying association between these hazard elements and epilepsy. Additionally, reverse MR analyses were performed to determine the causality of epilepsy on the association between these risk factors.

## Methods

### MR analysis

**Figure 1:**
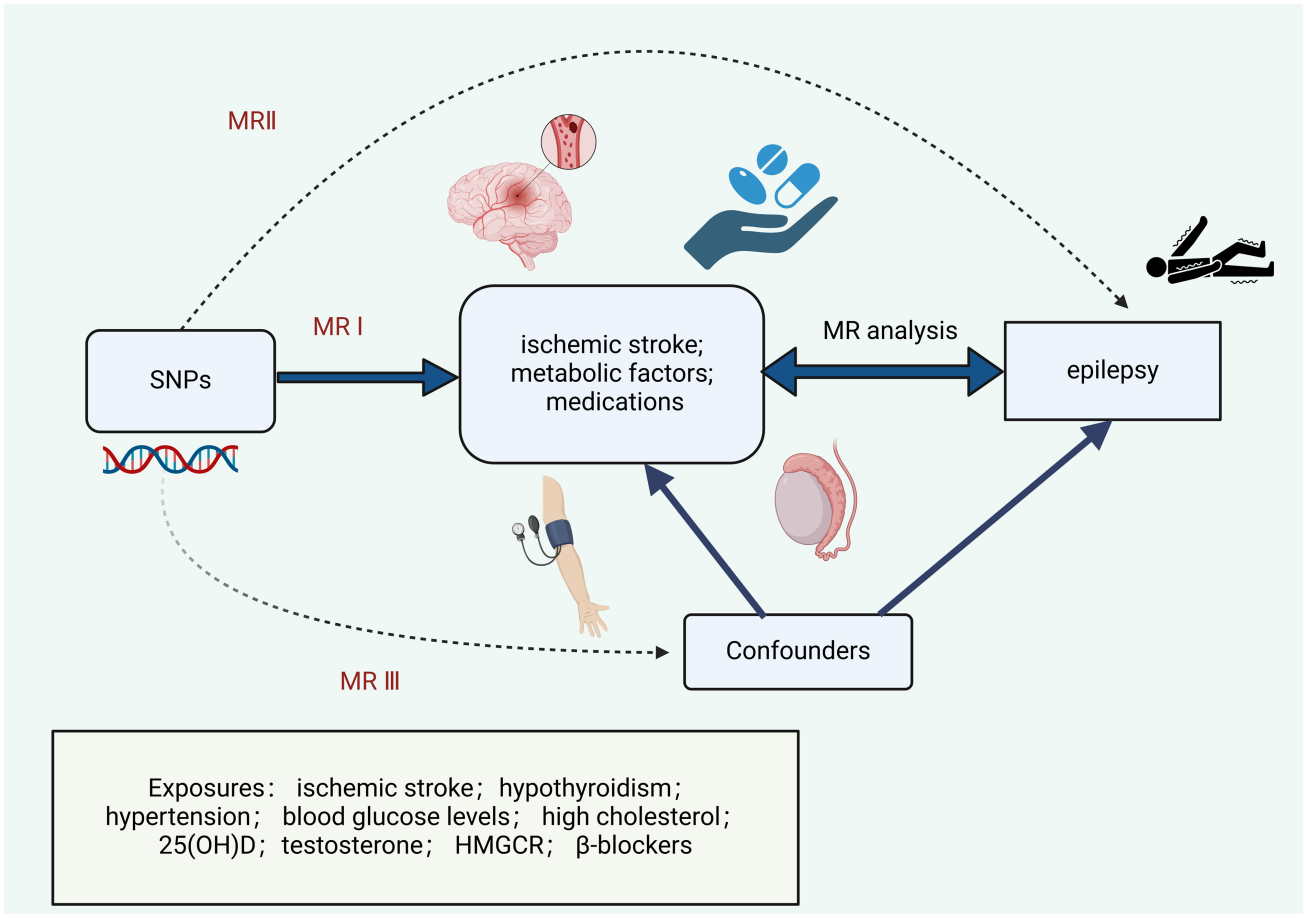
Research design and Mendelian randomization assumptions.

The flowchart of the MR analyses in this research is described in Figure 1. This research rigorously adheres to the STROBE-MR guidelines, incorporating the following three key assumptions (16): (A) single nucleotide polymorphism (SNP) is associated with ischemic stroke, hypothyroidism, hypertension, blood glucose levels, high cholesterol, 25(OH)D, testosterone, HMGCR, and *β*-blockers; (B) SNP influences epilepsy solely through ischemic stroke, hypothyroidism, hypertension, blood glucose levels, high cholesterol, 25(OH)D, testosterone, HMGCR, and β-blockers; (C) SNP is not linked to confounding factors.

### Data source

The data analyzed in the research were sourced from publicly available GWAS datasets, eliminating the need for additional moral permission. The GWAS pooled statistics encompassed various conditions, including epilepsy (*n* = 407,746), ischemic stroke (34,217 cases/ 406,111 controls), hypothyroidism (*n* = 405,357), high blood pressure (*n* = 407,746), blood glucose levels (*n* = 400,458), high cholesterol (*n* = 407,746), 25(OH)D (*n* = 417,580), high cholesterol (*n* = 407,746), HMGCR (73,475 cases/ 216,910 controls), beta blocking agents (*n* = 31,700 cases/ 192,324 controls) from the IEU Open GWAS project (17).^1^ The GWAS dataset for Testosterone is sourced from the UK Biobank (Table 1).

**Table 1:** Characteristics of genome-wide association study (GWAS) data.

Type	Traits	Source	GWAS ID	Ancestry	Sample size
Outcome	Epilepsy	IEU	ebi-a-GCST90013945	European	407,746
Exposure	Ischemic stroke	IEU	ebi-a-GCST005843	European	34,217 cases/ 406,111 controls
Exposure	Hypothyroidism or myxoedema	IEU	ebi-a-GCST90013893	European	405,357
Exposure	High blood pressure	IEU	ebi-a-GCST90013966	European	407,746
Exposure	Blood glucose levels	IEU	ebi-a-GCST90025986	European	400,458
Exposure	High cholesterol	IEU	ebi-a-GCST90013932	European	407,746
Exposure	Serum 25-Hydroxyvitamin D levels	IEU	ebi-a-GCST90000614	European	417,580
Exposure	Testosterone	UK Biobank	ukb-d-30850_irnt	European	13,585,069 SNPs
Exposure	HMG CoA reductase inhibitors	IEU	ebi-a-GCST90018989	European	73,475 cases/ 216,910 controls
Exposure	beta blocking agents	IEU	ebi-a-GCST90018986	European	31,700 cases/ 192,324 controls

### Selection of instrumental variables

We employed the following guidelines for selecting appropriate SNPs as IVs (18). Initially, a *p*-value significance threshold of 5 × 10–8 was set for SNPs. Subsequently, SNPs were pruned for linkage disequilibrium based on criteria of r^2^ < 0.001 and kb = 10,000. Next, the F-statistic was computed to evaluate the strength of the IVs, with SNPs having F-statistic values below 10 considered weak IVs and thus eliminated (Supplementary Table S1). Lastly, palindromic SNPs were excluded. Additionally, PhenoScanner was utilized to eliminate SNPs associated with confounding factors (19).

### Statistical analysis

We principal employed the inverse variance weighting (IVW) approach to analyze the causality between exposure risk and epilepsy. Significance was determined when *p* < 0.05. Additionally, supplementary techniques comprising the simple mode, MR-Egger, weighted median, and weighted mode were utilized. Heterogeneity was evaluated using Cochrane’s Q-test, while multiple validity analyses were performed utilizing MR-Egger and MR-PRESSO, with significance set at *p* < 0.05 for indicating heterogeneity and horizontal pleiotropy. All statistical analyses were performed utilizing the ‘TwoSampleMR’ package in R 4.3.2.

## Results

**Figure 2:**
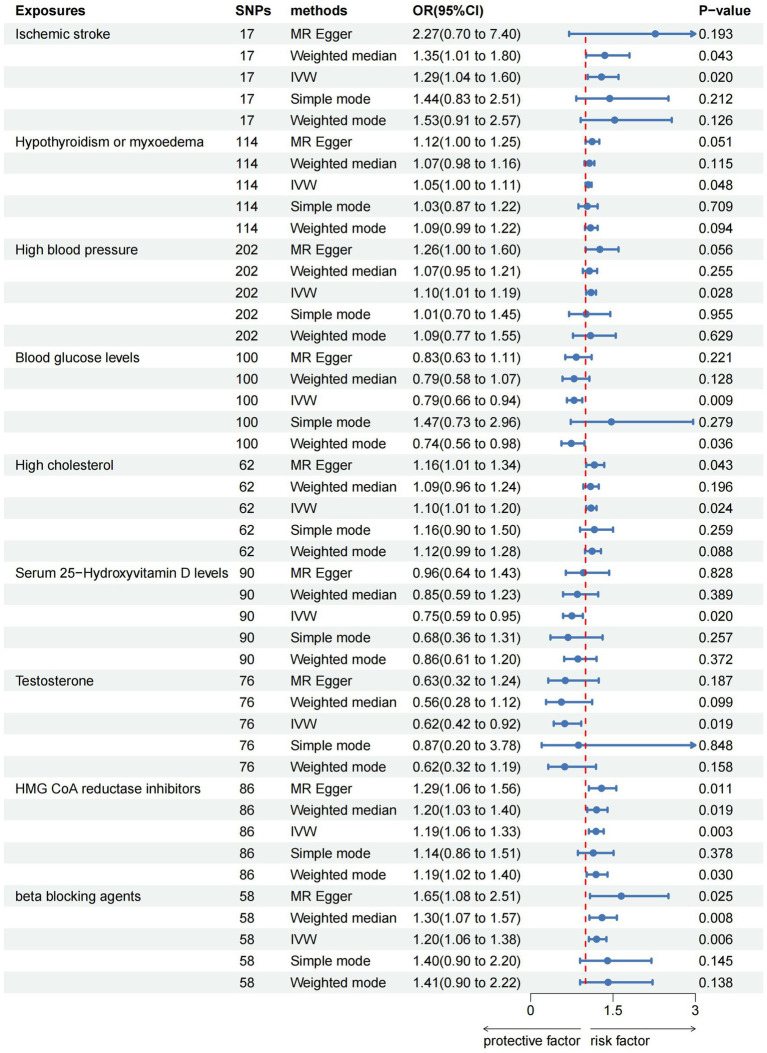
Forest plots of the causal relationship of ischemic stroke, metabolic factors, and related medications with epilepsy in the result of IVW in the MR analysis.

**Figure 3:**
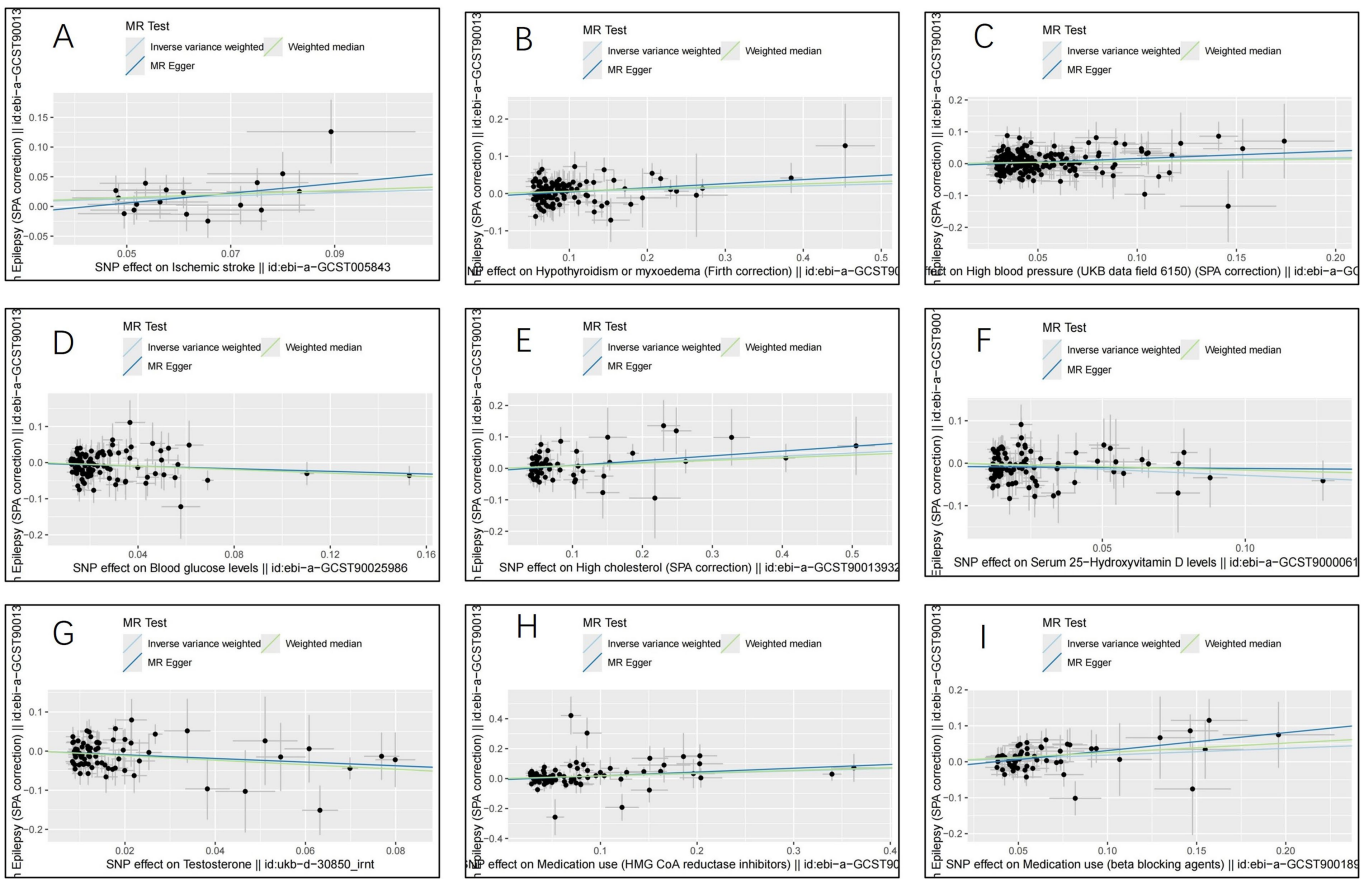
Scatter plots for the causal relationship of ischemic stroke, metabolic factors, and related medications with epilepsy. (A) ischemic stroke; (B) Hypothyroidism; (C) High blood pressure; (D) Blood glucose levels; (E) High cholesterol; (F) Serum 25 − Hydroxyvitamin D levels; (G) Testosterone; (H) HMG CoA reductase inhibitors; (I) beta blocking agents.

**Figure 4:**
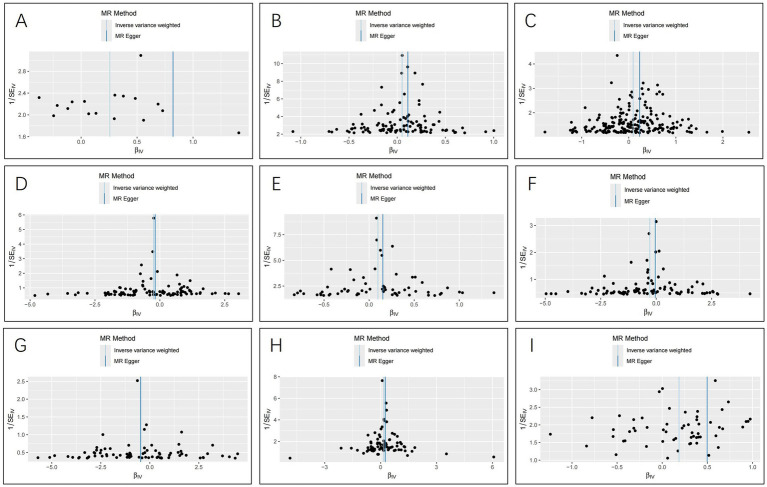
Funnel plots for the causal relationship of ischemic stroke, metabolic factors, and related medications with epilepsy. (A) ischemic stroke; (B) Hypothyroidism; (C) High blood pressure; (D) Blood glucose levels; (E) High cholesterol; (F) Serum 25 − Hydroxyvitamin D levels; (G) Testosterone; (H) HMG CoA reductase inhibitors; (I) beta blocking agents.

The IVW analysis indicated that ischemic stroke (OR = 1.29, 95% CI 1.04–1.60; *p* = 0.020), hypothyroidism (OR = 1.05, 95% CI 1.00–1.11; *p* = 0.048), high blood pressure (OR = 1.10, 95% CI 1.01–1.19; *p* = 0.028), high cholesterol (OR = 1.10, 95% CI 1.01–1.20; *p* = 0.024), HMGCR (OR = 1.19, 95% CI 1.06–1.33; *p* = 0.003), and beta-blocking agents (OR = 1.20, 95% CI 1.06–1.38; *p* = 0.006) are associated with an increased risk of epilepsy. Conversely, blood glucose levels (OR = 0.79, 95% CI 0.66–0.94; *p* = 0.009), 25(OH)D (OR = 0.75, 95% CI 0.59–0.95; *p* = 0.020), and testosterone (OR = 0.62, 95% CI 0.42–0.92; *p* = 0.019) exhibited negatively related to the risk of epilepsy (Figure 2). However, the IVW analysis indicated that no causal association between age (*p* = 0.737), sex (*p* = 0.231), and epilepsy. Sensitivity analyses confirmed the robustness of these results. The results of the MR sensitivity analysis are presented in Table 2. During the heterogeneity test, all *p*-values derived from Cochrane’s Q statistic were found to be greater than 0.05, indicating a lack of heterogeneity among the SNPs. Additionally, the MR-Egger regression intercept, used to assess horizontal pleiotropy, did not indicate the presence of pleiotropy. The MR-PRESSO results also confirmed the absence of significant horizontal pleiotropy outliers (*p* > 0.05). Leave-one-out analysis did not suggest that any individual SNPs had a significant impact on the overall results. Detailed results of the leave-one-out analysis and single forest plots are shown in Supplementary Figures S1–S2. As depicted in the scatter plot in Figure 3, no evidence of horizontal pleiotropy was detected in the MR-Egger regression (*p* > 0.05). The funnel plot in Figure 4 showed no apparent bias, further confirming the robustness of our findings. However, according to Supplementary Table S2, the *p* value for IVW was >0.05 or each method showed an inconsistent direction, thus reverse MR analysis showed no causal relationship between epilepsy and these several risk factors. Additionally, sensitivity analyses confirmed the robustness of our findings (Supplementary Table S3).

**Table 2:** MR sensitivity analysis results.

Exposures	Analytical method	OR(95%CI)	Q	Q_pval	egger_intercept_P	MR-PRESSO_P
Ischemic stroke	MR Egger	2.27(0.70,7.40)	13.92	0.685		
	IVW	1.29(1.04,1.60)	12.84	0.685	0.355	0.705
Hypothyroidism or myxoedema	MR Egger	1.12(1.00,1.25)	94.49	0.883		
	IVW	1.05(1.00,1.11)	95.91	0.876	0.235	0.879
High blood pressure	MR Egger	1.26(1.00,1.60)	173.35	0.914		
	IVW	1.10(1.01,1.19)	174.85	0.909	0.221	0.909
Blood glucose levels	MR Egger	0.83(0.63,1.11)	97.19	0.504		
	IVW	0.79(0.66,0.94)	97.48	0.524	0.591	0.539
High cholesterol	MR Egger	1.16(1.01,1.34)	60.34	0.464		
	IVW	1.10(1.01,1.20)	61.18	0.469	0.362	0.487
Serum 25-Hydroxyvitamin D levels	MR Egger	0.96(0.64,1.43)	87.86	0.484		
	IVW	0.75(0.59,0.95)	90.07	0.448	0.141	0.468
Testosterone	MR Egger	0.63(0.32,1.24)	81.56	0.256		
	IVW	0.62(0.42,0.92)	81.56	0.283	0.962	0.293
HMG CoA reductase inhibitors	MR Egger	1.29(1.06,1.56)	106.62	0.048		
	IVW	1.19(1.06,1.33)	107.98	0.047	0.305	0.058
Beta blocking agents	MR Egger	1.65(1.08,2.51)	44.70	0.861		
	IVW	1.20(1.06,1.38)	47.00	0.825	0.135	0.823

## Discussion

Our MR study investigated the causal association between nine previously identified risk factors and epilepsy. The study revealed that ischemic stroke, hypothyroidism, high cholesterol, hypoglycemia, high blood pressure, HMGCR usage, and beta-blockers usage are related to an increased risk of epilepsy. Conversely, higher levels of 25(OH)D and testosterone were found to be associated with a decreased risk of epilepsy. Future attention should be given to screening for epileptogenesis risk in the management of patients with ischemic stroke, hypothyroidism, high cholesterol, hypoglycemia, hypertension, low levels of 25(OH)D and testosterone, and those on long-term HMGCR inhibitors and *β*-blockers. Regular screening and monitoring may be necessary to facilitate early detection and intervention, potentially improving the overall prognosis for these patients.

The heightened risk of epilepsy following ischemic stroke has been documented in numerous prior cohort studies (20, 21). Nevertheless, establishing a causality between ischemic stroke and epilepsy still challenging due to the susceptibility of observational studies to other confounding factors and reverse causation. Our MR study revealed that each standard unit increase in ischemic stroke elevated the risk of epileptogenesis by 29%, aligning with findings from previous research. The onset of seizures shortly after ischemic stroke may be attributed to localized ionic displacement and the release of elevated levels of excitotoxic neurotransmitters in ischemic injury sites. Conversely, epilepsy that emerges gradually during later stages may stem from potentially permanent lesions resulting from sustained neuronal excitability seizures (22–24).

Metabolic disorders have been identified as a significant factor in epileptogenesis (25). Thyroid hormones not only regulate energy metabolism but also play roles in neuronal survival, differentiation, and central nervous system energy expenditure. Several studies have demonstrated links between thyroid function and neurological disorders like dementia and depression (26). Moreover, thyroid hormones play an essential role in the pathophysiology of epilepsy (6). Our research indicates that hypothyroidism elevates epilepsy risk. Primarily, insufficient thyroid hormones due to hypothyroidism can slow neuronal metabolism and disrupt neurotransmitter synthesis and release (27, 28). Secondly, hypothyroidism can cause electrolyte imbalances like hyponatremia, which affect brain electrical activity and raise seizure risk (29–31). Prolonged hypothyroidism may also induce structural changes in specific brain regions, such as white matter damage, further increasing seizure susceptibility (32, 33).

A longitudinal study indicates that high blood pressure escalates the risk of late-onset epilepsy by 2 to 2.5 times, consistent with our MR analysis findings (7). Hypertension can trigger seizures through both direct and indirect pathways. The renin-angiotensin system may serve as a pivotal link between hypertension and epilepsy (34). Elevated blood pressure might contribute to cerebrovascular diseases such as cerebral infarction and hemorrhage, leading to brain damage, ischemic and hypoxic dysfunction, white matter lesions, and disruption of normal neuronal function, consequently heightening seizure susceptibility (35, 36). Furthermore, hypertension-induced systemic and localized inflammatory responses, along with oxidative stress products, can damage neurons and alter neuronal excitability, potentially inducing seizures (37, 38).

Prior studies consistently indicate that chronic glucose metabolism disorders frequently correlate with long-term epileptogenesis (39). Our research suggests that hypoglycemia increases the risk of epilepsy. The human brain heavily depends on glucose for energy, extracting sufficient amounts from the bloodstream to sustain normal functions. Persistent hypoglycemia triggers neuronal necrosis due to oxygen deprivation, subsequently leading to abnormal discharges capable of inducing seizures (40–43).

Disorders in brain cholesterol metabolism have been related to various neurological conditions like Parkinson’s disease, Alzheimer’s disease, and epilepsy (44–46). Our research indicates that each standard unit increase in cholesterol elevates epilepsy risk by 10 percent. Cholesterol might indirectly trigger epilepsy by impacting blood vessels, leading to cerebrovascular diseases and subsequently epilepsy. Excessive cholesterol accumulation, a crucial brain component, can not only increase membrane viscosity and decrease extrasynaptic neurotransmitter receptor mobility but also directly regulate numerous voltage-dependent and ligand-gated ion channels, thus heightening excitotoxicity and focal neuronal death (47). This process may contribute to neurological complications following prolonged epilepsy. Additionally, neurosteroids have been proposed to influence the clinical course of epileptic disorders by modulating neurotransmission (48, 49).

The relationship between vitamin D deficiency and epilepsy has been extensively researched (50). Researches have indicated that correcting vitamin D deficiency can lead to improvements in seizures (51). While previous MR analyses have not identified a causality between 25(OH)D and epilepsy, our MR analysis suggests that each standard unit increase in 25(OH)D reduces seizure risk by 25% (52). Vitamin D, a steroid hormone, plays an important role in regulating calcium homeostasis, neuroprotection, and brain function and development. The activated form of vitamin D, 25(OH)D, is primarily implicated in seizures. The predominant mechanism in current seizure research involves an unbalance between GABAergic inhibitory signaling and glutamatergic excitatory signaling at the synapse. Activation of voltage-gated calcium channels is vital for neuronal processes like neurotransmitter release, excitation, and synaptic transmission. Deficiency in 25(OH)D decreases the expression of these channels, leading to increased calcium ions in neurons and subsequent production of nitric oxide (NO)-dependent neuronal nitric oxide synthase, resulting in oxidative stress damage to the neuronal endoplasmic reticulum (53). Therefore, 25(OH)D may confer neuroprotection by reducing hyperexcitability in epileptic patients through improvements in calcium and magnesium levels and by attenuating oxidative damage to cells via NO inhibition (54–56).

The association between sex steroid hormones and epilepsy is a topic of significant interest and has undergone extensive study (12). Nevertheless, the causality between hormones and epilepsy still somewhat ambiguous. Our MR analysis revealed that each standard deviation decrease in testosterone levels decreased the risk of epilepsy by 38%, lending crucial theoretical support to previous studies exploring testosterone therapy for epilepsy (57). There are several mechanisms through which testosterone may exert its antiepileptic effects. Firstly, testosterone exhibits neuroprotective properties and can modulate neural activity by reducing glutamate release and enhancing GABA function (58). Secondly, testosterone has been shown to mitigate neuroinflammation, thereby reducing the frequency and severity of seizures (59). Additionally, testosterone can influence neuronal excitability by modulating ion channel function, enhancing neuronal membrane stability, and decreasing the likelihood of neuronal discharge (60). Reduction of oxidative stress may also facilitate to the antiepileptogenic effects of testosterone (61). Finally, testosterone may reduce seizure risk by affecting neurotransmitter balance in the brain, thereby preserving normal neuronal function (62).

Many drugs have the potential to induce epilepsy, yet this type of epilepsy is often overlooked by clinicians. The mechanisms behind drug-induced seizures are diverse and may include direct effects on the central nervous system, electrolyte imbalances, metabolic disturbances, and more (63). HMGCR, an important statin, has primarily been associated with anticonvulsant effects in previous studies. However, our research revealed that HMGCR may actually increase the risk of epilepsy, possibly due to its ability to induce hypokalemia, a known seizure trigger (64). Additionally, HMGCR might interact with other antiepileptic medications, affecting their efficacy (65). HMGCR inhibitors may influence the plasma concentration of certain antiepileptic drugs by competing for their metabolic pathways, potentially altering their efficacy or increasing the risk of adverse effects. Additionally, HMGCR inhibitors might indirectly affect neuronal membrane stability by altering cholesterol metabolism, which could influence the seizure threshold. Moreover, the anti-inflammatory effects of statins may interact synergistically or antagonistically with the actions of specific antiepileptic drugs. Moreover, HMGCR may cross the blood–brain barrier and directly influence the central nervous system, potentially leading to seizures (66, 67). Past reports have also documented cases of seizures induced by beta receptor antagonists (68). Hypoglycemic seizures are a commonly reported serious adverse effect of propranolol (69). Our MR analysis demonstrated that each standard unit increase in beta-blocking agent administration raised the risk of epileptogenesis by 20%. Consequently, we propose that beta-blocking agents can induce epilepsy by inducing hypoglycemia and interfering with the efficacy of other antiepileptic drugs. Beta-blockers may potentially impact the efficacy of antiepileptic drugs by reducing sympathetic nervous system activity and altering neurotransmitter balance in the brain. In certain cases, beta-blockers may indirectly influence the metabolism and clearance of antiepileptic drugs by affecting blood flow to the liver or kidneys. These changes could lead to lower plasma concentrations of the antiepileptic drugs, thereby reducing their effectiveness. Beta-blockers cause hypoglycemia probably through direct inhibition of hepatic production of glucose and release of glucagon. In addition, by attenuating the counter-regulatory effects of adrenaline, thereby promoting sympathetic-induced glycogenolysis and reducing gluconeogenesis. However, an analysis showed differences between short- and long-half-life beta blockers (particularly nadolol) and non-selective and selective beta blockers in terms of the risk of hypoglycemia occurring (70). Studies have shown that the risk of hypoglycemia appears to be higher with the use of non-selective beta blockers and long-acting beta blockers.

While our MR study offers a thorough examination of the causal relationship between various risk factors, including ischemic stroke, metabolic factors, and associated medications, with epilepsy, it is vital to admit some limitations. Firstly, the predominance of GWAS data from European ethnic populations calls for caution when generalizing our findings to other ethnic groups. Secondly, due to the aggregated nature of our data, we lacked access to detailed individual-level information for further stratified analyses. Additionally, although MR minimizes concerns regarding reverse causality and confounding factors, there may still exist some residual biases that could potentially impact the reliability of our results.

## Conclusion

In summary, our study indicates that ischemic stroke, hypothyroidism, hypertension, high cholesterol, hypoglycemia, HMGCR inhibitors, and *β*-blockers may elevate the risk of epilepsy, whereas high levels of 25(OH)D may decrease the risk. These findings offer valuable insights for the tertiary prevention of epilepsy in clinical settings and suggest potential avenues for further research into the mechanisms underlying epilepsy.

## Data Availability

The original contributions presented in the study are included in the article/Supplementary material, further inquiries can be directed to the corresponding author.

## Footnotes

^1^ https://gwas.mrcieu.ac.uk/
