# Supplementary material for: Causal associations of ischemic stroke, metabolic factors, and related medications with epilepsy: a Mendelian randomization study
Source: Front Neurol. 2024 Nov 13;15:1464984. doi: 10.3389/fneur.2024.1464984 (PMC11598930; doi:10.3389/fneur.2024.1464984)
Supplement: Supplementary file 1 [file Supplementary_file_1.docx]

**Supplementary Figure S1:** Leave-one-out plots for the causal relationship of ischemic stroke, metabolic factors, and related medications with epilepsy.


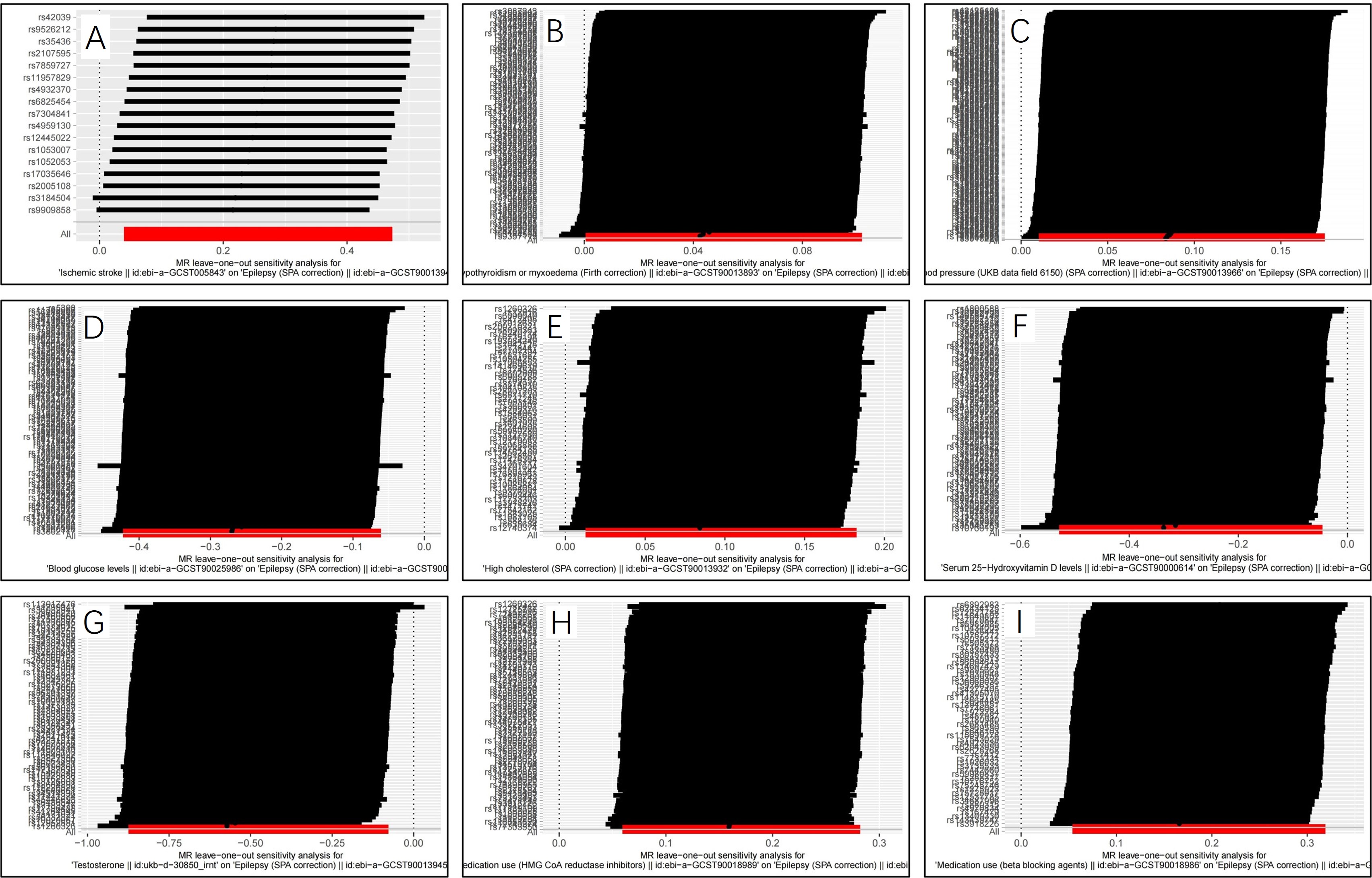


1. ischemic stroke (B) Hypothyroidism (C) High blood pressure (D) Blood glucose levels

(E) High cholesterol (F) Serum 25−Hydroxyvitamin D levels (G) Testosterone (H) HMG CoA reductase inhibitors (I) beta blocking agents
