# Supplementary material for: Causal associations of ischemic stroke, metabolic factors, and related medications with epilepsy: a Mendelian randomization study
Source: Front Neurol. 2024 Nov 13;15:1464984. doi: 10.3389/fneur.2024.1464984 (PMC11598930; doi:10.3389/fneur.2024.1464984)
Supplement: Supplementary file 4 [file Table_2.docx]

**Supplementary Table S2**: The causal relationship of epilepsy on ischemic stroke, metabolic factors, and related medications in the result of IVW in the reverse MR analysis.

| **Outcome** | **Method** | **nSNP** | **b** | **se** | **OR (95%CI)** | **pval** |
| --- | --- | --- | --- | --- | --- | --- |
| ischemic stroke | IVW | 10 | -0.01 | 0.03 | 0.99(0.93,1.06) | 0.771 |
| Hypothyroidism or myxoedema | IVW | 13 | -0.01 | 0.02 | 0.99(0.95,1.04) | 0.717 |
| High blood pressure | IVW | 13 | 0.04 | 0.02 | 1.04(1.00,1.08) | 0.048 |
| Blood glucose levels | IVW | 4 | 0.02 | 0.02 | 1.02(0.98,1.06) | 0.379 |
| High cholesterol | IVW | 13 | 0.01 | 0.02 | 1.01(0.96,1.06) | 0.708 |
| Serum 25-Hydroxyvitamin D levels | IVW | 11 | 0.01 | 0.01 | 0.78(0.63,0.95) | 0.848 |
| Testosterone | IVW | 12 | -0.01 | 0.01 | 1.00(0.99,1.01) | 0.493 |
| HMG CoA reductase inhibitors | IVW | 11 | 0.03 | 0.02 | 1.03(0.99,1.08) | 0.148 |
| beta blocking agents | IVW | 10 | 0.02 | 0.08 | 1.02(0.98,1.06) | 0.428 |
