# Supplementary material for: Causal associations of ischemic stroke, metabolic factors, and related medications with epilepsy: a Mendelian randomization study
Source: Front Neurol. 2024 Nov 13;15:1464984. doi: 10.3389/fneur.2024.1464984 (PMC11598930; doi:10.3389/fneur.2024.1464984)
Supplement: Supplementary file 5 [file Table_3.docx]

**Supplementary Table S3: Reverse MR sensitivity analysis results.**

| Outcome | Analytical method | OR (95%CI) | Q | Q_pval | egger_intercept_P | MR-PRESSO_P |
| --- | --- | --- | --- | --- | --- | --- |
| Ischemic stroke | MR Egger | 0.85(0.72,1.01) | 10.45 | 0.235 |  |  |
|  | IVW | 0.99(0.93,1.06) | 15.14 | 0.087 | 0.095 | 0.100 |
| Hypothyroidism or myxoedema | MR Egger | 0.98(0.89,1.09) | 10.35 | 0.499 |  |  |
|  | IVW | 0.99(0.95,1.04) | 10.39 | 0.581 | 0.838 | 0.591 |
| High blood pressure | MR Egger | 0.96(0.89,1.03) | 19.57 | 0.052 |  |  |
|  | IVW | 1.04(1.00,1.08) | 29.79 | 0.003 | 0.035 | 0.006 |
| Blood glucose levels | MR Egger | 1.00(0.90,1.10) | 12.21 | 0.002 |  |  |
|  | IVW | 1.02(0.98,1.06) | 13.42 | 0.004 | 0.700 | 0.034 |
| High cholesterol | MR Egger | 0.93(0.84,1.03) | 19.05 | 0.060 |  |  |
|  | IVW | 1.01(0.96,1.06) | 24.59 | 0.017 | 0.101 | 0.017 |
| Serum 25-Hydroxyvitamin D levels | MR Egger | 1.00(0.95,1.05) | 24.32 | 0.004 |  |  |
|  | IVW | 1.00(0.99,1.02) | 24.37 | 0.007 | 0.900 | 0.008 |
| Testosterone | MR Egger | 1.01(0.99,1.03) | 10.23 | 0.420 |  |  |
|  | IVW | 1.00(0.99,1.01) | 13.25 | 0.277 | 0.117 | 0.292 |
| HMG CoA reductase inhibitors | MR Egger | 0.93(0.83,1.04) | 19.17 | 0.024 |  |  |
|  | IVW | 1.03(0.99,1.08) | 26.98 | 0.003 | 0.088 | 0.006 |
| beta blocking agents | MR Egger | 0.87(0.77,0.99) | 2.61 | 0.956 |  |  |
|  | IVW | 1.02(0.98,1.06) | 9.36 | 0.405 | 0.032 | 0.424 |
